# A machine learning model to estimate myocardial stiffness from EDPVR

**DOI:** 10.1038/s41598-022-09128-6

**Published:** 2022-03-31

**Authors:** Hamed Babaei, Emilio A. Mendiola, Sunder Neelakantan, Qian Xiang, Alexander Vang, Richard A. F. Dixon, Dipan J. Shah, Peter Vanderslice, Gaurav Choudhary, Reza Avazmohammadi

**Affiliations:** 1 Department of Biomedical Engineering, Texas A&M University, College Station, TX 77843 USA; 2 Department of Molecular Cardiology, Texas Heart Institute, Houston, TX 77030 USA; 3 Providence VA Medical Center, Providence, RI 02908 USA; 4 Houston Methodist DeBakey Heart and Vascular Center, Houston, TX 77030 USA; 5 Department of Medicine, Alpert Medical School of Brown University, Providence, RI 02903 USA; 6 J. Mike Walker ’66 Department of Mechanical Engineering, Texas A&M University, College Station, TX 77843 USA; 7 Department of Cardiovascular Sciences, Houston Methodist Academic Institute, Houston, TX 77030 USA

**Keywords:** Cardiovascular models, Biomedical engineering

## Abstract

In-vivo estimation of mechanical properties of the myocardium is essential for patient-specific diagnosis and prognosis of cardiac disease involving myocardial remodeling, including myocardial infarction and heart failure with preserved ejection fraction. Current approaches use time-consuming finite-element (FE) inverse methods that involve reconstructing and meshing the heart geometry, imposing measured loading, and conducting computationally expensive iterative FE simulations. In this paper, we propose a machine learning (ML) model that feasibly and accurately predicts passive myocardial properties directly from select geometric, architectural, and hemodynamic measures, thus bypassing exhaustive steps commonly required in cardiac FE inverse problems. Geometric and fiber-orientation features were chosen to be readily obtainable from standard cardiac imaging protocols. The end-diastolic pressure-volume relationship (EDPVR), which can be obtained using a single-point pressure-volume measurement, was used as a hemodynamic (loading) feature. A comprehensive ML training dataset in the geometry-architecture-loading space was generated, including a wide variety of partially synthesized rodent heart geometry and myofiber helicity possibilities, and a broad range of EDPVRs obtained using forward FE simulations. Latin hypercube sampling was used to create 2500 examples for training, validation, and testing. A multi-layer feed-forward neural network (MFNN) was used as a deep learning agent to train the ML model. The model showed excellent performance in predicting stiffness parameters $a_f$ and $b_f$ associated with fiber direction ($R^2_{a_f}=99.471\%$ and $R^2_{b_f}=92.837\%$). After conducting permutation feature importance analysis, the ML performance further improved for $b_f$ ($R^2_{b_f}=96.240\%$), and the left ventricular volume and endocardial area were found to be the most critical geometric features for accurate predictions. The ML model predictions were evaluated further in two cases: (i) rat-specific stiffness data measured using ex-vivo mechanical testing, and (ii) patient-specific estimation using FE inverse modeling. Excellent agreements with ML predictions were found for both cases. The trained ML model offers a feasible technology to estimate patient-specific myocardial properties, thus, bridging the gap between EDPVR, as a confounded organ-level metric for tissue stiffness, and intrinsic tissue-level properties. These properties provide incremental information relative to traditional organ-level indices for cardiac function, improving the clinical assessment and prognosis of cardiac diseases.

## Introduction

Alteration of myocardial wall stiffness is known to be a key myocardial remodeling mechanism in many cardiac diseases, including heart failure with reduced and preserved ejection fraction. Non-invasive techniques to assess myocardial stiffness can facilitate early and precise diagnosis of structural heart disease and advance patient-specific treatment to reduce the incidence and mortality of heart diseases^1^. Cardiac imaging modalities, including cardiac magnetic resonance (CMR) and computed tomography (CT), as well as hemodynamic assessment via invasive catheterization and non-invasive echocardiography indices, provide a large amount of data primarily describing the cardiac anatomy and function at the organ level^2^. These organ-level measurements provide important geometric and boundary condition information required for patient-specific cardiac inverse-FE modeling to estimate cardiac properties at smaller length scales including tissue and fiber. Despite significant advances in such modeling paradigms in the past 10 years, their translation to clinical use remains limited, particularly due to the exhaustive efforts required to prepare and conduct these models. Therefore, there remains an unmet need to create a predictive, yet clinically feasible, modeling tool to infer myocardial properties from standard cardiac imaging and functional assessment modalities.

Many early studies of myocardial biomechanics consisted of ex-vivo mechanical testing of fresh myocardial tissue to quantify passive behavior of the myocardium^3–5^. These studies provided a deep understanding of highly nonlinear and anisotropic behavior of myocardial tissues prompting several constitutive modeling efforts to effectively capture the passive behavior of myocardium in 3D^6,7^. The ex-vivo mechanical testing, once conducted properly, may serve as “ground-truth” measurements for any in-vivo estimated myocardial properties that often rely on integrated imaging-inverse FE modeling to deliver properties in an ideally non-invasive manner^8–12^. With the goal of estimating passive properties, the inverse problem is often built to minimize the difference between the end diastolic pressure volume relationship (EDPVR), measured through catheterization, and the EDPVR that is generated through imaged-based FE modeling using pressure-controlled boundary conditions. The minimization problem involves searching through the constitutive parameter space (using gradient-based^13^ or genetic algorithm methods) and iteratively adjusts the error until the objective function falls below the target error. Such an FE inverse problem typically involves the following steps leading to the optimal constitutive parameters: (1) reconstruction of the heart geometry using imaging data and meshing, (2) prescription of constitutive modeling as the material model, (3) recovering an unloaded state from a known loaded state (e.g. end-diastole)^14^, and (4) setting up a nonlinear optimization problem and iteratively fine-tuning the parameters to matches the target EDPVR. As described, the typical pipeline to conduct inverse-FE simulations^15–18^ is quite exhaustive, involving steps from image segmentation to iterative inverse simulation, and often taking a few days with high-performance computing resources to yield the desired results. Expectedly, such computational and processing demands hamper the feasibility of this approach in time-sensitive clinical applications.

Recently, machine learning (ML) techniques, particularly deep learning^19^, have emerged as a promising tools^20–23^ to fill the gap between traditional FE simulations and the realization of the inverse modeling approach in imaging-driven clinical cardiovascular applications. Several studies to replace FE simulations with ML surrogate models have already been conducted for vascular applications. For instance, Liang et al. studied the feasibility of deep learning to estimate the steady-state distributions of pressure and flow velocity inside the thoracic aorta^24^. They also used deep learning to predict the zero-pressure geometry^25^ and stress distribution^26^ of the human thoracic aorta in separate works. In these works, Liang et al. used FE analysis to generate training dataset. Also, Liu et al. proposed a multilayer neural networks to estimate in-vivo constitutive parameters of aortic wall^27^. The application of ML in enhancing patient-specific cardiac modeling has recently gained increased interest as well. Dabiri et al. used eXtreme Gradient Boosting (XGBoost) and Cubist^28^ as well as feed-forward and a recurrent neural network with long short-term memory^29^ to predict left ventricle (LV) pressures, volumes and stresses. Cai et al.^30^ developed a ML surrogate model that maps the material parameter features to low-dimensional parameterized expressions for LV pressure-volume and pressure-strain relations. Despite these emerging advances in synergizing ML and FE cardiac modeling, these studies have primarily focused on demonstrating the feasibility, reliability, and accuracy of ML surrogate models in replacing *forward*-FE simulations with various ML approaches. There remains a critical need to investigate how *inverse*-FE simulations to estimate patient-specific cardiac characteristics, typically the end goal of cardiac modeling applications, could benefit from ML surrogate models. Moreover, recent studies have focused on the applications of ML when the heart geometry and/or microstructure is fixed, while the ultimate need to produce a ML surrogate that could deliver predictions for new cardiac anatomy and architecture still remains.

In this paper, we present a novel and feasible ML model to estimate patient-specific myocardial stiffness in the LV *in-vivo* using the LV-EDPVR. The model is intended to replace the costly and exhaustive FE approach to predict myocardial properties using the EDPVR as input. We designed and trained our ML model on a large and partially synthetic dataset of rodent heart geometry, fiber orientation, and loading response (EDPVR). The model is able to predict myocardial properties directly from input features that includes limited information about heart geometry, fiber data, and EDPVR. The geometric features were chosen such that they can be easily measured using standard CMR and cardiac CT protocols. Rat-specific geometries were used to generate a large and partially synthesized database of biventricular examples. A broad range of fiber architectures and material properties were created. The Latin hypercube sampling (LHS)^31^, as a statistical method for generating a near-random sample of parameter values from a multidimensional distribution, was used for effective coverage of parameter space and avoiding clustered sample points. The EDPVR for each example was computed using forward-FE simulations to complete inputs and outputs of our supervised ML model. Relevant parameters for the geometry, fiber orientation, and material properties were sampled using LHS. A multilayer feed-forward neural network (MFNN) was trained as an *inverse model* to predict myocardium stiffness as an output given geometric features, fiber orientation, and EDPVR as the inputs. The trained and tested ML model was examined further to predict myocardial properties for new rodent hearts and compared against rat-specific ex-vivo “ground-truth” measurements of the properties. Finally, our ML model was used to predict myocardial properties in a human heart with cardiac MRI data.

## Materials and methods

### Animal models

A total of 25 male rats were used in this study. The group included nine Sprague-Dawley, nine Fischer-344, and seven Wistar Kyoto rats. The cohort consisted of healthy rats (n=8), rats with pulmonary hypertension (PH; n=11), and rats with myocardial infarction (MI; n=6). The hypertensive rats were of Sugen5416-hypoxia model of PH^32^, and the infarcted rats developed MI via ligation of the left anterior descending artery^33^. All experiments were carried out in accordance with relevant guidelines and regulations as described in the Methods section. The surgical and experimental protocols for the MI rats were approved by Institutional Animal Care and Use Committee (IACUC) at the Texas heart institute (Protocol 2020-0023). The surgical and experimental protocols for the PH rats were approved by IACUC at the Providence VA Medical Center (Protocol 2019-009). The methods used for animal research in this study have been reported according to the Animal Research: Reporting of In Vivo Experiments (ARRIVE) guidelines.

### Image acquisition and reconstruction

In this study, high-resolution anatomical magnetic resonance imaging (MRI) scans were performed on the harvested hearts using a Bruker Biospec 7T (Billerica, MA) scanner. The hearts were fixed approximately at end-diastole shape^5^ and the scans were performed at an isotropic 60 $\mu$m resolution (FOV = 15 $\times$ 15 $\times$ 20 mm, $T_R$ = 40 ms, and $T_E$ = 7.5 ms). The segmentation and reconstruction of 3-D full heart geometry from the MRI scans were performed using the Mimics Innovation Suite (Materialise, Leuven, Belgium). Epicardial and endocardial surfaces were isolated from reconstructed geometries to create a library of epicardial and ventricular surfaces. Surfaces were used to expand our geometry database for ML training . All the geometries were truncated below the valve plane and meshed using quadratic tetrahedral elements.

### Machine learning model versus FE inverse model

The estimation of myocardial stiffness (as tissue-level properties) from the EDPVR (as an organ-level input) depends on the geometry of the heart and fiber architecture in the myocardium. We propose a ML approach that maps the input space including geometry, fiber orientation, and EDPVR features to the output, namely myocardial properties, based on the example input-output pairs in the training dataset. Contrary to the time-consuming, image-based inverse FE method that, for each given heart, needs to be set up and conducted separately to determine myocardial properties, we present a ML model trained by FE-produced input-output pairs that can determine the myocardial properties for each new heart in a split second only through limited, and commonly available, input data on geometry, fiber orientation, and EDPVR.

**Figure 1:**
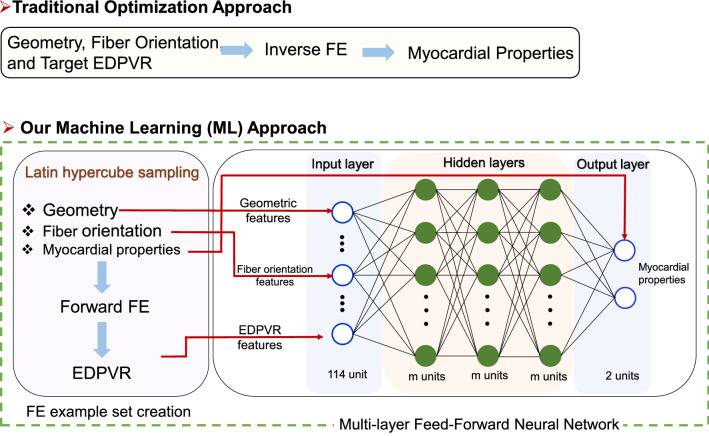
The flowchart for traditional inverse FE approach versus the proposed ML approach.

The workflow for our proposed ML approach has two main parts (Fig. 1): (i) creating a complete input and output space (left panel in Fig. 1), and (ii) using MFNN as a deep learning agent to train ML on sampled input-output pairs (right panel in Fig. 1). The creation of geometry, architecture, and EDPVR input space is described in detail in the “ML input features and sampling” subsection and the MFNN training and testing is detailed in the “Deep learning model” subsection. Here, it is important to note that we advantageously trained our ML inverse model on forward-simulated FE input-output pairs *without* solving an inverse FE problem. Indeed, myocardial properties were first considered as “known” input to create EDPVR on sampled (geometry, architecture, myocardial properties space). After sampling and creation of all the required EDPVR curves using forward FE simulations, each calculated EDPVR was grouped with their respective geometric and architectural counterparts and the corresponding properties were taken as *output* to produce proper input-output examples for ML for supervised learning. The myocardial properties parameter space (output space) and geometry, architecture, and EDPVR database (input space) are described next.

### Myocardial material properties

The myocardium is an anisotropic soft tissue exhibiting a nonlinear and exponential-like stress-strain behavior within the physiological range of deformation. It is often modeled as a hyperelastic incompressible solid characterized by the stored energy function $\Psi (\mathbf{C})$ with $\mathbf {C}$ being the right Green-Lagrange strain tensor. A reduced version of structurally based constitutive model proposed by Holzapfel and Ogden^34^ was considered in this work representing the myocardium as a transversely isotropic solid with the local preferred direction $\mathbf{f}_0$^35^. The resulting energy function thus takes the form

1 $$\begin{aligned} \Psi = \frac{a}{2b}exp[b(I_1-3)]+\frac{a_f}{2b_f}\{exp[b_f(I_{4f}-1)^2]-1\} \end{aligned}$$

where *a*, *b*, $a_f$ and $b_f$ are four positive material constants, *a* and $a_f$ have stress dimension whereas *b* and $b_f$ are dimensionless. Also, the kinematics $I_1$ and $I_{4f}$ are defined as $I_1=tr\;\mathbf{C}$ and $I_{4f}=\mathbf{f}_0{\cdot }(\mathbf{C}\,\mathbf{f}_0)$ which are intended to capture the deformation in the ground matrix and along the myofiber direction, respectively. Using standard notation, the resulting Cauchy stress tensor, defined as ${\varvec{\sigma }}=\mathbf{{F}}\,{\partial \psi }/{\partial \mathbf {F}}-p\, \mathbf {I}$, is given by

2 $$\begin{aligned} \varvec{\sigma }=a\; exp[b(I_1-3)]\mathbf{B}-p\,\mathbf{I}+2a_f(I_{4f}-1)exp[b_f(I_{4f}-1)^2]\mathbf{f}\otimes \mathbf{f} \end{aligned}$$

where *p* is the hydrostatic pressure, $\mathbf {B}=\mathbf {F}\, \mathbf {F}^T$ is the left Cauchy-Green tensor, $\mathbf {F}$ is the deformation gradient, $\mathbf{f}=\mathbf{F}\,\mathbf{f}_0$, and $\mathbf {I}$ is the identity tensor. The incompressiblity constraint $J=det \; \mathbf{F}=1$ is enforced.

**Figure 2:**
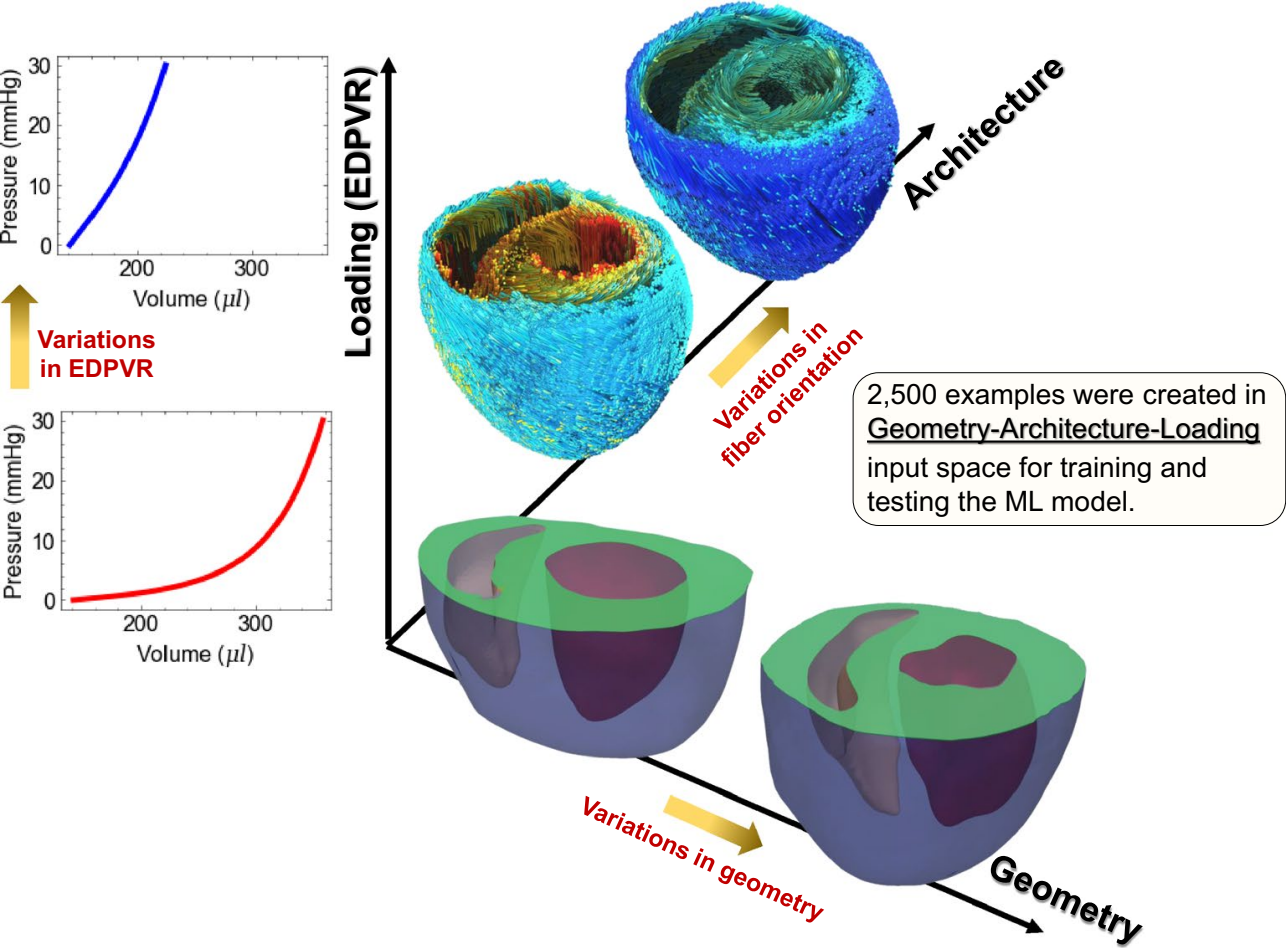
ML model feature space including geometry, fiber architecture, and loading (EDPVR).

Our pilot inverse-FE estimation of the constants $\{a,\ b,\ a_f,\ b_f\}$ from EDPVR indicated that *a* and $a_f$, representing the ground matrix and fiber stiffness, respectively, have a strong correlation and make, quantitatively different, but qualitatively similar contributions to the EDPVR (an example is detailed in the “Myocardial parameter identifiability”) subsection. Such correlations were noted in previous studies^2,8,36^ considering the full version of the H–O model and addressed by (independent) parameter reduction.

Therefore, to improve parameter identifiability in our inverse problem and, given our interest in estimating myofiber properties and a low contribution of the ground matrix to myocardial mechanical behavior^37^, we set $a=0.22$ kPa and $b=1.62$ as reference values for the ground matrix in healthy LV myocardium^30,38^ and let $\{a_f,\ b_f\}$ to be the only material parameter variables in this work. Indeed, our preliminary inverse model studies verified that $\{a_f,\ b_f\}$ can be uniquely calculated using an EDPVR input.

### ML input features and sampling

Our input space consisted of EDPVR, geometry, and architecture (Fig. 2). Using LHS for sampling, we produced 2,500 input-output examples. In the following subsections, geometry and architecture input spaces and their respective features are separately discussed followed by the details of our sampling method and the generation of EDPVR curve to be used as input along with the geometry and architecture.

#### Geometry database and feature extraction

**Figure 3:**
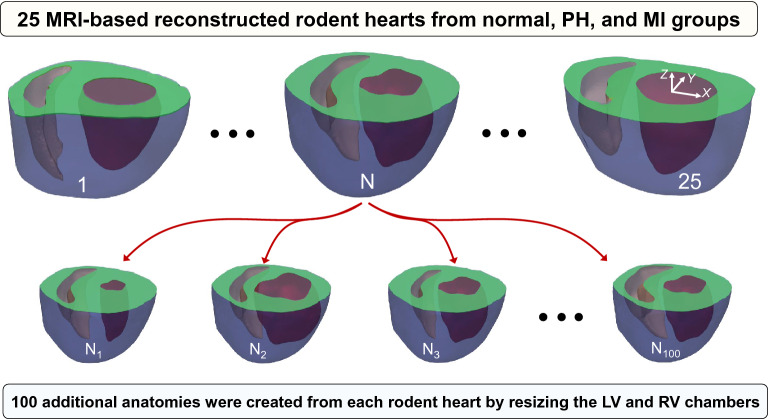
Creation of partially synthetic heart geometries using MRI-based reconstructed biventricular hearts.

Each of 25 MRI-based reconstructed geometries, referred to as “parent” hearts, were used to create additional geometries via resizing the LV and right ventricle (RV) endocardial surfaces (Fig. 3) using an automated workflow. The chambers were resized isotropically in *X-Y* plane while maintaining the center of area on the base (truncated) plane. The epicardial surface was kept fixed in each “parent” heart. The resizing factors, denoted by constants $f_{LV}$ and $f_{RV}$ for the left and right ventricles, respectively, were obtained using LHS discussed in the “Sampling method: LHS” subsection. Our automated workflow led to the creation of 2,500 different geometries.

**Figure 4:**
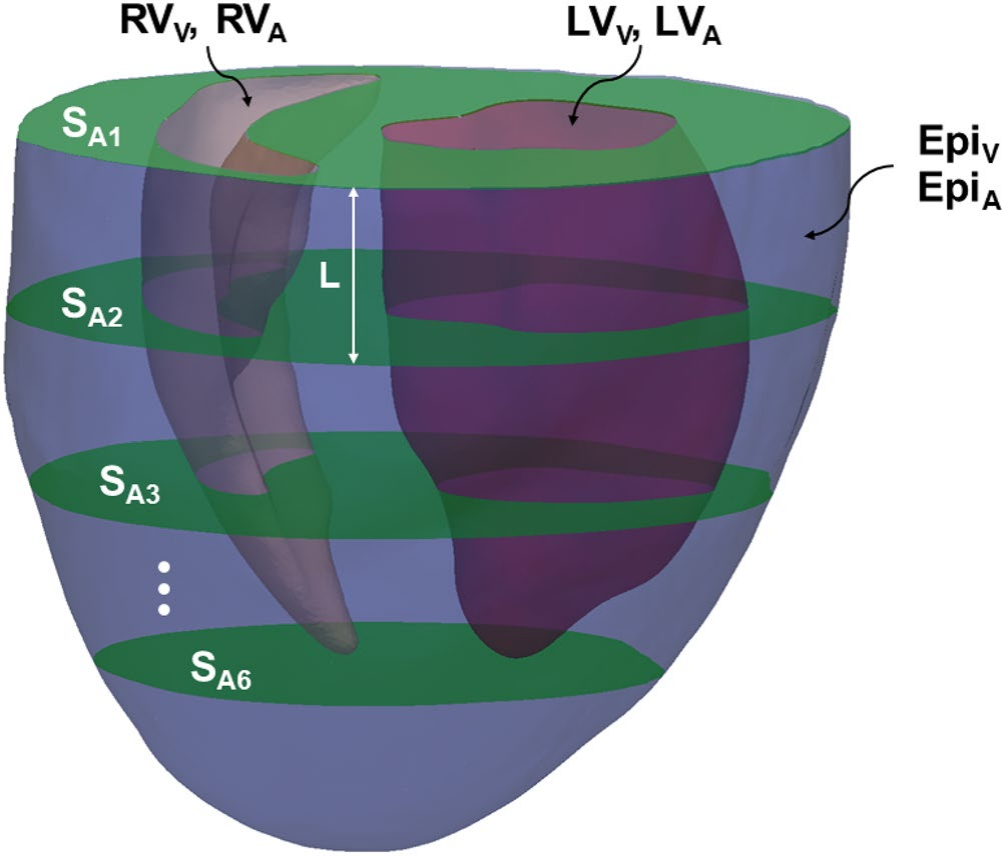
Geometric features: Volume and endocardial area of the LV ($LV_V$ and $LV_A$), volume and endocardial area of the RV ($RV_V$ and $RV_A$), interior volume and area of the epicardial surface ($Epi_V$ and $Epi_A$), and the area of six, equally-spaced, short-axis slices ($S_{A1}, S_{A2},..., S_{A6}$).

The role of heart geometry is significant in the estimation of myocardial properties from an organ-level measurement (i.e., EDPVR) and any proposed geometric features should capture this role. Moreover, motivated by clinical applications of the proposed ML, the geometric features need to be compatible with the routine in-vivo scanning protocols of the heart using common cardiac imaging modalities, including CMR, CT, and echocardiography. Given these considerations, we propose to begin with the following 12 features for the geometry (Fig. 4): volume and endocardial area of the LV chamber, denoted by $LV_{V}$ and $LV_{A}$, volume and endocardial area of the RV chamber, denoted by $RV_{V}$ and $RV_{A}$, the area of six, equally-spaced, short-axis slices from the base plane towards the apex, denoted by $S_{A1}$ to $S_{A6}$ with $S_{A1}$ denoting the basal slice, and finally the interior volume and the area of the epicardial surface, denoted by $Epi_{V}$ and $Epi_{A}$. These features were designed to be feasibly acquired via CMR acquisitions. We calculated these features using reconstructed geometries; however, they can be calculated using only segmented slices reducing the post-processing needed to quantify the geometric features.

#### Fiber architecture

All the geometries were meshed and fiber orientations were assigned to each FE model using Laplace-Dirichlet rule-based algorithm^39^. The algorithm involves solving several Laplace-Dirichlet boundary value problems to determine the relative depth of each tetrahedral element and its local material axes within the myocardial wall. All the myocardial walls in our anatomies were divided to four depths (layers) and each layer was assigned a scalar identifying its fiber orientation relative to the circumferential direction. The fiber helicity was assumed to linearly turn from a positive value at endocardium towards a negative value at epicardium. Positive and negative angular values within $(-\pi /2,\ \pi /2)$ at endo- and epicardium, denoted by $\theta _{endo}$ and $\theta _{epi}$, respectively, were chosen as two features characterizing myocardial architecture. All possible values were considered (given the sign constraint at endo- and epicardial surfaces) in LHS sampling.

#### Sampling method: LHS

LHS^31^ was used to generate a near-random sample of parameter values from a multidimensional distribution. LHS can effectively avoids issues associated with points being clustered together, occurring in other commonly used sampling methods such as grid sampling or uniform sampling. Briefly, sampling for six features $f_{LV},\ f_{RV},\ \theta _{endo},\ \theta _{epi},\ a_f,\ b_f$, the range of each each was divided into 2,500 equally probable intervals. Next, 2,500 sample points were chosen to satisfy the Latin hypercube constraint preventing any two sample points to share the same feature. That is, all the 2,500 examples had different geometric, architectural, and loading features. In this study, out of 2,500 samples chosen by LHS, 2,400 examples were used for training and validation and the remainder for testing.

As mentioned in the “Machine learning model versus FE inverse model” subsection, LHS was applied to the (geometry, architecture, myocardial properties) parameter space, and the resulting samples were used to generate EDPVR curves. In turn, the generated data were re-grouped to produce desired input-output (geometry, architecture, EDPVR - myocardial properties) pairs for ML training. The limits of introduced features for sampling were set as $0.7<f_{LV}<1.6$, $0.7<f_{RV}<1.6$, $0^{\circ }<\theta _{endo}<\pi /2$, $-pi/2<\theta _{epi}<0^\circ$, $0.01<a_f<100$ (kPa), and $0.01<b_f<20$. The resizing factors ranges were chosen to ensure the LV and RV resizing without the intersection of surfaces. The fiber angles ranges assured a negative helical slope from endocardium to epicardium. And, the ranges for myocardial properties were chosen to produce a wide variety of EDPVR possibilities based on EDPVR examples in Klotz et al.^40^.

#### EDPVR

The EDPVR was used as the input to estimate passive properties of the myocardium. The EDPVR was calculated for each sampled geometry-architecture-properties using forward FE simulations in ABAQUS (Simulia, Providence, RI, USA). The basal surface was fixed in the long-axial direction (*Z*). A linearly ramped pressure from 0 to 30 mmHg was applied to the LV endocardial surface with 100 equal loading steps. The pressure in the RV was considered to be 20% of LV pressure. The volume of the LV chamber was recorded in each of the 100 loading increments, and the recorded points served as 100 EDPVR features.

### Deep learning model

The proposed machine learning task in this study is a supervised learning regression problem for which a strongly nonlinear relation between input features and the output is expected. Although several regression methods such as regular nonlinear regression, decision-tree ensembles e.g. XGBoost, and K-nearest neighbour (KNN), could be used for this problem, the multilayer perceptron method MFNN was chosen for the following three reasons: (i) our hybrid image-based-synthetic dataset includes several thousands examples identifying as a large dataset for which deep learning algorithms are known to outperform most machine learning methods^41^, (ii) deep learning has proven as an effective tool to identify very complex nonlinear relationships between input and outputs^42^ compared to most machine learning methods, and (iii) deep learning offers thorough and automated feature engineering serving as a crucial step in developing effective machine learning models. Concerning the last point, feature engineering is performed manually in traditional machine learning methods, excessively depending on the knowledge of domain experts, whereas neural networks benefit from intrinsic automated feature engineering capability through automatically transforming the original features into a set of new features^43^.

Since our data to be learned is neither sequential nor time-dependent, a MFNN was used as a nonlinear supervised learning module. That is, information travels forward through the perceptron units in the network (no loops are permitted) to compute a nonlinear function *f* on fixed-size input ${\varvec{x}}$ such that $f({\varvec{x}}) \approx {\varvec{y}}$ for training pairs $({\varvec{x}},{\varvec{y}})$. Each perceptron unit with n-dimensional input ${\varvec{z}}$ and n-dimensional weight ${\varvec{w}}$ and bias *b*, and activation function $g({\varvec{u}})$ has a 1-dimensional output $o=g({\varvec{w}}\varvec{.}{\varvec{z}}+b)$. The input layer had 114 units which includes 12 geometric features, two fiber orientation features, and 100 EDPVR features (volume values collected at 100 equally spaced points on EDPVR). We used two hidden layers with 512 units, and finally the output layer representing the two material properties $\{a_f,\ b_f\}$. TensorFlow version 2.4.0 was used as the deep learning training library.

#### Error estimation and cross-validation

The inputs were standardized removing the mean and scaling to unit variance, however, outputs were normalized using the maximum absolute value of each dimension. These choices were based on our preliminary explorations of the normalization and the prediction assessment. After training, the predicted material properties were rescaled to their original range. We used mean absolute error (MAE) as the loss function, defined as

3 $$\begin{aligned} MAE=\frac{1}{n}\sum _{i=1}^2\sum _{j=1}^n \left| \bar{y}_i^{(j)}-\hat{\bar{y}}_i^{(j)}\right| , \end{aligned}$$

where $\bar{y}$ and $\hat{\bar{y}}$ are actual and predicted normalized material parameters, respectively, and *n* is the number of data points (input-output pairs). The subscript *i* denotes the *i*th material parameter and superscript (*j*) is the index for input-output pair.

The discrepancy between actual and predicted material parameters are quantified by normalized mean absolute error (NMAE). The NMAE of the *i*th material parameter is defined as

4 $$\begin{aligned} NMAE_i=\frac{\sum _{j=1}^n \left| y_i^{(j)}-{\hat{y}}_i^{(j)}\right| }{n (max(y_i^{(j)})-min(y_i^{(j)}))} \end{aligned}$$

We also calculated the coefficient of determination, $R^2$, as

5 $$\begin{aligned} R^2_i=1-\frac{\sum _{j=1}^n \left( y_i^{(j)}-{\hat{y}}_i^{(j)}\right) ^2}{\sum _{j=1}^n \left( y_i^{(j)}-\tilde{y}_i\right) ^2}; \quad \tilde{y}_i=\frac{1}{n}\sum _{j=1}^{n}y_i^{(j)} \end{aligned}$$

Adaptive moment estimation (Adam) method was used for the optimization. The rectified linear unit (ReLu), $g(x)= x^+ =max(0,x)$, was used for the activation function. The ML model was trained on training/validation dataset and the trained ML model was used to predict the material parameters in the testing dataset. We used 2,400 examples for training and validation dataset, and 100 examples for testing dataset.

Performance of the deep learning model was evaluated using a ten-fold cross-validation approach. In each step of the cross-validation, $90\%$ of training/validation data points were randomly selected as the training data and the remaining $10\%$ were used for validation. Number of layers and units in each layer were adjusted in the ranges of 1 to 3 and 64 to 1,024, respectively, to minimize the average NMAE in the cross-validation step. To prevent overfitting, early stopping was performed to secure optimized results at epochs with minimum validation error. In the cross-validation step, 2,000 epochs were performed with 0.0001 learning rate, and 10,000 epochs were performed for final training and predictions on testing dataset, once the network structure was tuned.

### Comparisons with ex-vivo mechanical test measurements

To further evaluate our ML model, we compared the myocardium stiffness predicted by ML for one new healthy male rat heart against the ground-truth stiffness obtained from ex-vivo equibiaxial tension test of the harvested LV free wall (LVFW) specimen. Short-axis echocardiography images was performed using a Philips iE33 Echocardiography machine (Bothell, WA,98021, United States) with a S12-4 transducer (12 MHz). Rat-specific pressure-volume (P-V) measurements were obtained at the terminal point using a rat-size P-V catheter (1.4F, Millar Instruments Inc., Houston, Texas). Geometric features were approximated from the combination of short-axis images and catheter-based volume measurements (Supplementary Fig. 1). Following a similar approach as in Klotz et al.^40^, the EDPVR data was generated using $$(P_{ED}$$, $$V_{ED})$$ data point obtained from the P-V loop. Briefly, a universal EDPVR curve was created using 2,500 training examples in the “ML input features and sampling” subsection using the normalization approach described in Klotz et al.^40^ (Supplementary Fig. 2). The universal EDPVR was used to generate the individual EDPVR for the new rat using $$(P_{ED}$$, $$V_{ED})$$. The transmural fiber orientation was measured using histological sectioning parallel to the epicardial surface. The angles at four depths were quantified as $$1.06^\circ , -12.88^\circ , -20.31^\circ $$, and $$-21.48^\circ $$ in the LVFW from endo- to epicardium, respectively (Supplementary Fig. 3). Equibiaxial tension test along longitudinal and circumferential directions was used to characterize the in-plane mechanical behavior of the LVFW^44^. The trace of the first Piola-Kirchhoff (1st PK) stress tensor $$\mathbf {P}$$, i.e., $$P_{11}+P_{22}$$ where $$\mathbf{P}=J\,{\varvec{\sigma }}\,\mathbf{F}^{-T}$$, measured from biaxial tests as the function of the stretch was fit to the respective stress expression using Eq. (2), and doing appropriate algebra, to calculate the constants $$\{a_f,\ b_f\}$$ with $$a=0.22$$ kPa and $$b=1.62$$ being kept constant as before. The ML model predictions for $$\{a_f,\ b_f\}$$ were obtained using the quantified geometric features, fiber orientations, and the EDPVR, and compared against $$\{a_f,\ b_f\}$$ obtained using equibiaxial testing.

### Application to human heart

**Figure 5:**
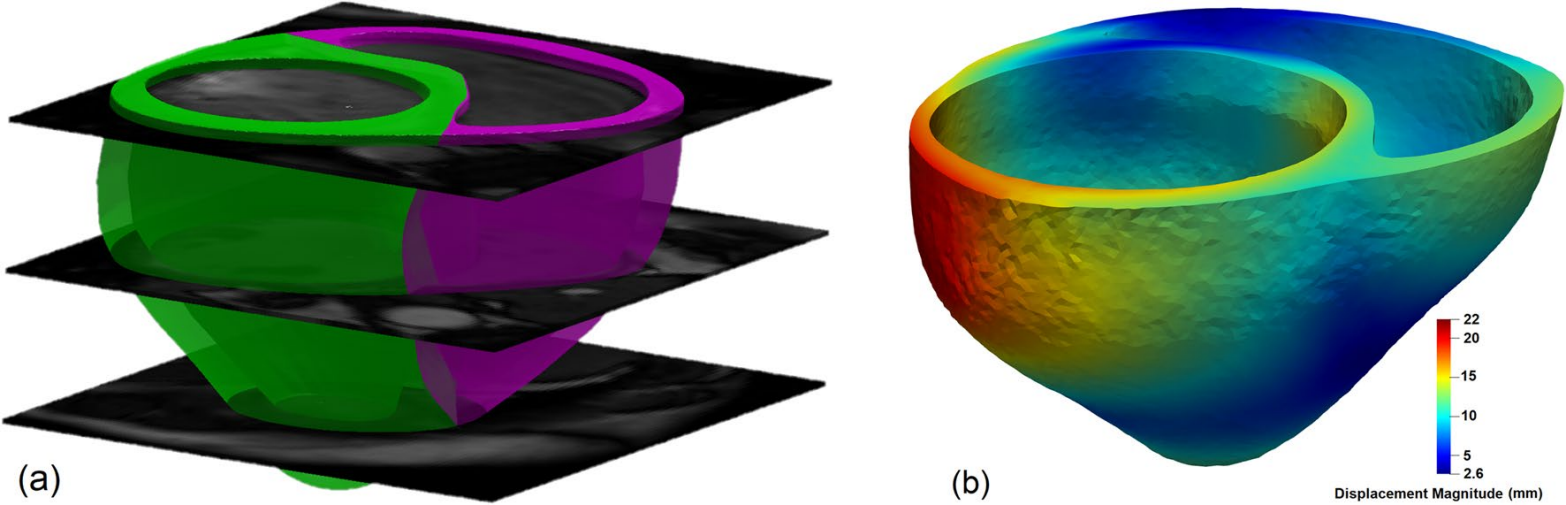
Application of the ML model to human heart: (**a**) anatomy reconstruction at ED using CMR scans (**b**) a representative displacement field generated in the inverse FE problem.

We used input from a human heart and compared the predictions of the trained ML model for myocardial properties against those from an inverse FE simulation. The data belonged to a patient with mitral valve prolapse (MVP) and mitral valve regurgitation. Cine CMR scans were acquired using a 3.0-T clinical scanner (Siemens Verio; Siemens, Erlangen, Germany) with phased-array coil systems. A standard CMR examination for mitral valve assessment consisted of a cine-CMR for anatomic and functional assessment in a short axis stack using a steady-state free-precession sequence with typical flip angle of 65-85 degrees; repetition time of 3.0 ms; echo time of 1.3 ms; in-plane spatial resolution of 1.7–2.0 $\times$ 1.4–1.6 mm; slice thickness of 6 mm with 4 mm interslice gap; and temporal resolution of 35–40 ms. CMR scans were used to reconstruct the heart geometry at ED (Fig. 5a).

The geometric features (Fig. 4) were quantified from the reconstructed anatomy. Since diffusion tensor imaging was not performed for the subject, the fiber orientation angles were set to $+60^\circ$ and $-60^\circ$ at endo- and epicardial surfaces, respectively, and vary linearly in between, as typically assumed in cardiac modeling of the human heart^45^. The EDPVR curve was obtained using the Klotz-like universal curve discussed in the rodent example (Supplementary Fig. 2) and the P–V data point at ED. The volume at ED was estimated from the reconstructed anatomy and $P_{ED}=18$ mmHg was used for the pressure reported as a mean value for the ED pressure in the LV in MVP patients^46^. The resulting EDPVR together with geometric and architectural features were used in the ML model to predict myocardium material properties. For comparison purposes, the properties were predicted using the commonly used inverse FE method and the Levenberg-Marquardt algorithm for optimization (Fig. 5b). A negative $P_{ED}$ was applied to approximate the unloaded geometry.

### Feature importance

Finally, the permutation feature importance technique^47^ was used to study the sensitivity of our ML model predictions of myocardial properties to different features. This technique serves as a model inspection method that can be used for any fitted estimator of tabular data. This approach is useful, particularly, for non-linear or opaque estimators such as MFNN. The permutation feature importance is quantified by the decrease in a model score (MAE) when a single feature value is randomly shuffled. This procedure breaks the relationship between the feature input and the true output, thus the drop in the model score is indicative of how much the model depends on the feature. The permutation feature importance is calculated many times with different permutations of the feature.

We used scikit-learn 0.24.2 library for the permutation importance. The technique is described in detail in Breiman et al.^47^, and a brief outline is provided here. The inputs used to calculate the feature importance are the trained ML model *m* and the tabular dataset (training/validation) *D*. The technique computes the reference score *s* (MAE) of the model *m* on data *D*. Next, for each feature $f_j$ (column of *D*) and for each repetition *k* (in 1,...,K), it randomly shuffles column *j* of dataset *D* to generate a corrupted version of the data named $\tilde{D}_{k,j}$. Next, it computes the score $s_{k,j}$ of model *m* on the corrupted data $\tilde{D}_{k,j}$. Finally, the importance $i_j$ for the feature $f_j$ is calculated as $i_j=s-\frac{1}{K}\sum _{k=1}^K s_{k,j}$. It is worth noting that features with low importance outcome for a model with a poor predictive power could be very important for a model with a high predictive power and vice versa. Therefore, we compute importances for the final structure once the best prediction power is reached. The results were in the same range when performed on training or validation datasets. Lastly, we note that permutation importance reflects how important a given feature is for *a particular predictive model* and may significantly vary from one model to another.

## Results

### Myocardial parameter identifiability

**Figure 6:**
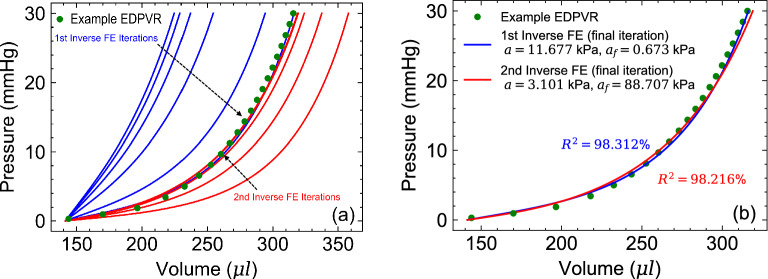
Constitutive parameter identifiability issue with using EDPVR as the target: (**a**) inverse FE model iterations to fit an example EDPVR starting from two different initial guesses for $\{a,\ a_f\}$ (**b**) Resulting fits at the final iteration leading to different values for $\{a,\ a_f\}$ with equally good fits. Parameters $b=b_f=5$ were kept constant during the optimization.

The correlations between the constitutive parameters model in Eq. (1), in the context of parameter estimation from EDPVR only, was examined first to select constitutive parameters with minimal correlations for optimization and preemptively eliminate potential identifiability issues in the ML model. A rat-specific anatomy with animal-specific diffusion-tensor-imaging-based architecture was used to conduct inverse FE optimization (Fig. 6a). An EDPVR curve was generated using the Klotz universal curve^40^ and the animal specific P-V data point ($V_{ED}= 140.5\;\upmu\text{l}$, $P_{ED}=20\; \text{mmHg}$) was used. Several preliminary FE inverse studies were performed to identify constitutive parameters with strongest correlations. A representative study showed a strong correlation between *a* and $a_f$ (Fig. 6).

The target EDPVR was reached through multiple iterations using two different sets of initial guesses for $\{a, a_f\}$ far apart in the parameter space (Fig. 6a). Despite distinctively different initial conditions, both optimizations successfully fit the target EDPVR with minimal errors (Fig. 6b). Expectedly, the optimizations predicted two completely different value sets for $\{a,\ a_f\}$ pairs: (11.677, 0.673) kPa versus (3.101, 88.707) kPa (Fig. 6b). This observation implied that *a* and $a_f$ could have very similar effects on the EDPVR and can not be uniquely determined using an EDPVR curve if taken as variable. On the contrary, the optimization with $\{a_f,\ b_f\}$ being set to vary (and *a* and *b* being constant) consistently led to a unique estimation of $\{a_f,\ b_f\}$ through fitting to the EDPVR for a broad range of different initial conditions. $a_f$ and $b_f$ found to have their dominant effects on the overall stiffness and toe-region of the EDPVR, respectively, hence, contributing differently to the EDPVR. Given this observations, $a_f$ and $b_f$ were chosen as material properties features for the ML model. $\{a,\ b\}$ were kept as described in the “Myocardial material properties” subsection for the remainder of our results.

### EDPVR variation

**Figure 7:**
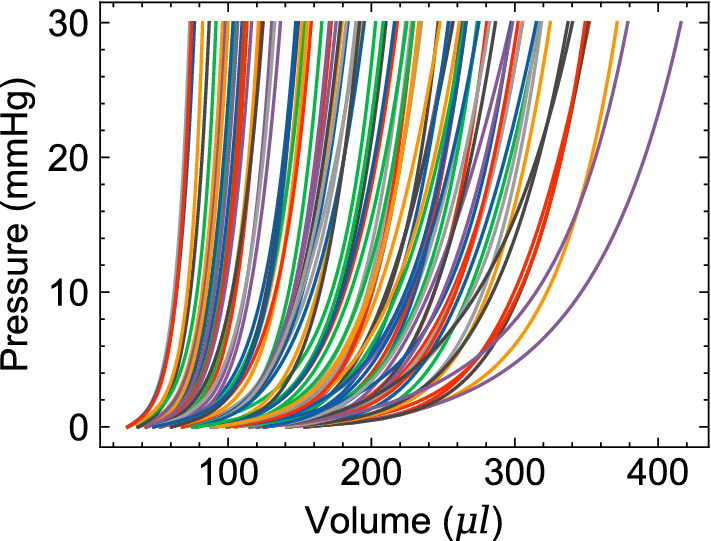
Representative EDPVRs (100 out of 2500) in ML training dataset resulting from a wide variety of heart geometries, fiber orientations, and myocardial stiffness.

A comprehensive database of the LV-EDPVR curves was generated (Fig. 7) by considering 2,500 combinations of geometry, fiber orientations, and material properties, selected by LHS as described in the “ML input features and sampling” subsection. The range of resizing for heart geometries were chosen such that the initial LV volume ($V_0$) varies from $\sim 35\;\mu$l up to about five times larger (Fig. 7), a range similar to the that of EDPVR curves studied in Klotz et al.^40^. The range included a broad range of soft to stiff LV chambers, as well as LV chambers with a short toe region to chambers with fibrotic myocardium, extending the toe-region, to severely diluted chambers, all enabled through changing $\{a_f,\ b_f\}$, fiber orientation, and the geometry.

### MFNN network structure

**Figure 8:**
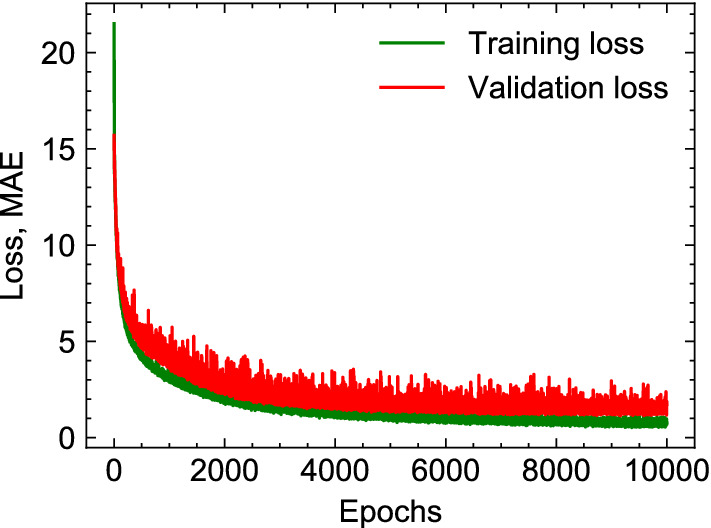
MAE loss function for training and validation datasets as a function of epochs.

We began with a single hidden layer with 64 units and iteratively changed the number of layers and units to find the optimal structure. Performance of different networks are summarized in Table 2 reporting MAE, NMAE, and $R^2$ scores for each examined structure. We found that a network with two hidden layers, each containing 1024 units, provides the best prediction, and it was chosen as the final structure. The MAE loss function for training and validation monotonically decreased with training epochs confirming that data was not over-fitted (Fig. 8), despite the fluctuations which are inevitable consequences of stochastic approach of mini-batch gradient descent in Adam (batch size=32). The model with the lowest validation loss was picked for predictions in testing dataset.

**Table 1:** ML prediction errors for testing dataset with final MFNN network structure and 10000 epochs.

	MAE	NMAE, $\%$	$R^2$, $\%$
$a_f$	1.326 (kPa)	1.336	99.471
$b_f$	0.963	5.008	92.837

**Table 2:** Averaged MAE, NMAE, and $R^2$ scores for two properties $a_f$ and $b_f$ with respect to network structure in ten-fold cross-validation (2000 epochs).

Network structure	MAE		NMAE, %		$R^2, \%$	
Hidden layer $\times$ unit	$a_f$ (kPa)	$b_f$	$a_f$	$b_f$	$a_f$	$b_f$
$1\times 64$	6.991	3.125	7.035	16.101	88.387	49.855
$1\times 128$	7.258	3.083	7.320	15.557	87.377	53.843
$1\times 256$	5.852	3.142	6.069	15.953	88.761	52.704
$1\times 512$	5.553	2.740	5.664	13.863	91.943	63.438
$1\times 1024$	5.444	2.353	5.523	11.857	91.120	67.213
$2\times 64$	5.193	2.627	5.219	13.335	93.749	68.647
$2\times 128$	3.100	2.073	3.152	10.765	97.606	74.895
$2\times 256$	2.499	1.708	2.550	8.645	98.426	84.317
$2\times 512$	2.214	1.281	2.228	6.486	98.395	89.331
$\mathbf {2\times 1024}$	**1.755**	**1.084**	**1.786**	**5.558**	**99.214**	**91.481**
$3\times 64$	4.505	2.225	4.613	11.164	94.505	75.674
$3\times 128$	2.498	1.572	2.614	8.002	97.754	84.299
$3\times 256$	1.958	1.238	1.981	6.350	98.888	89.948
$3\times 512$	2.313	1.324	2.343	6.742	98.388	88.298
$3\times 1024$	2.542	1.767	2.568	9.048	97.764	75.940

The 2 × 1024 structure had the best prediction and was used in the ML model.

### ML model prediction and performance

**Figure 9:**
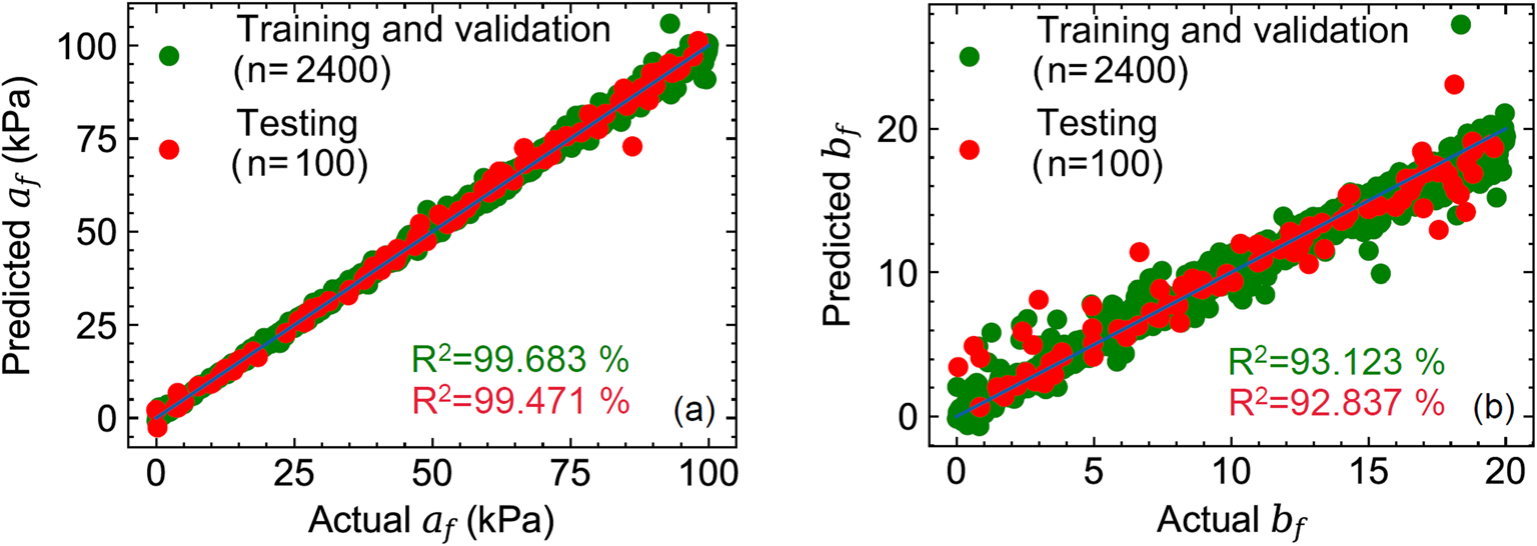
Actual and predicted values of myocardial properties: (a) $a_f$ (b) $b_f$. Training/validation and testing datasets included n=2400 and n=100 sample points, respectively.

Given the input features for the geometry, fiber orientations, and EDPVR, the trained ML model was able to predict the myocardial properties $\{a_f,\ b_f\}$ within one second on a PC with 2.8 GHz quad core CPU and 32 GB RAM. Prediction errors for testing dataset with final MFNN network structure and 10000 epochs showed low and acceptable errors in prediction (Table 1) using examples in the testing dataset that were not present in the training dataset. The actual versus predicted material parameters for training/validation and testing dataset for $a_f$ (Fig. 9a) and $b_f$ (Fig. 9b) indicated excellent agreements between ML predictions for both material parameters and their respective actual values. Predictions for $a_f$ were consistently better than that of $b_f$ ($R^2_{a_f}=99.5\%\ vs.\ R^2_{b_f}=92.8\%$; calculated for testing dataset). This is likely due the fact that the constant $b_f$ has a more dominant effect of the curvature of the input EDPVR for which the ML model need to learn the slope of EDPVR at several points whereas $a_f$ uniformly scales the EDPVR curve and can be predicted with a fewer points of the EDPVR.

**Figure 10:**
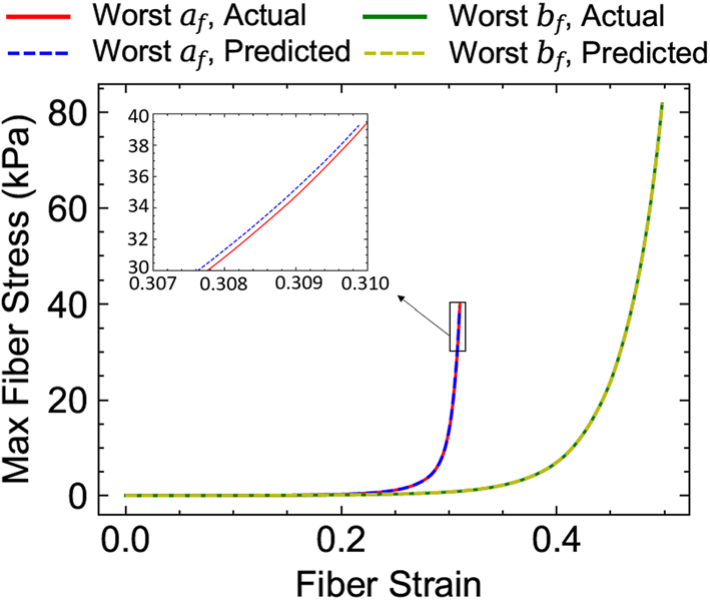
Maximum fiber stress in the LV free wall obtained by forward FE simulations using the predicted properties versus actual properties for the worst predictions listed in Table 3.

The ML-predicated myocardial properties for the best and worst predictions in terms of the MAE error in the entire testing dataset (Table 3) were used in forward FE simulations passively pressurizing the LV up to 30 mmHg (using respective geometries and fiber orientations). The resulting maximum stresses (along the fiber direction) in the LVFW as a function of fiber stain were compared against the corresponding quantities obtained using actual properties used as the input for testing (Fig. 10). The comparisons indicated that even the fiber stress (that is regarded as an important mechanical stimuli in regulating myocardial remodeling) associated with the worst predicted myocardial properties can still fit the actual stresses very well with $R^2>99.9\%$ (Fig. 10). This observation partly stems from the fact that $a_f$ and $b_f$ are somewhat correlated meaning that $\{a_f,\ b_f\}$ pairs with different individual values can still produce similar myocardial stress behavior, and that the ML model precisely reflects this feature of the material model. Therefore, the ML model performance can be still trusted for worst cases if the fiber stress is the indented target and not individual stiffness parameters.

**Table 3:** Predicted and actual values of myocardial properties for the best and worst predictions of $a_f$ and $b_f$ in the testing dataset (*n* = 100).

		$a_f$	$b_f$
Worst $a_f$	Actual	86.221	6.660
	Predicted	72.909	11.411
Best $a_f$	Actual	38.005	8.960
	Predicted	37.995	8.870
Worst $b_f$	Actual	85.771	2.990
	Predicted	85.020	8.130
Best $b_f$	Actual	92.815	17.450
	Predicted	95.160	17.434

**Table 4:** ML model prediction of rat-specific myocardial properties compared to estimates from ex-vivo equibiaxial testing of the LVFW myocardium specimen for the same rat.

Rat case study	$a_f\;(kPa)$	$b_f$
Equibiaxial test	0.806	0.470
ML model	0.583	0.521

**Figure 11:**
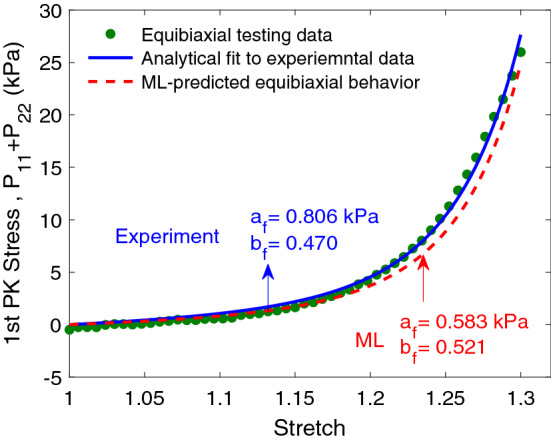
Stress-stretch response of rat LVFW myocardium under equibiaxial testing. The corresponding response predicted by the ML model is included for comparison.

### ML prediction comparisons with equibiaxial test results

Experimental and ML-predicted properties $a_f$ and $b_f$ were in very good agreement with the corresponding properties obtained from equibiaxial experiments (Table 4). The equibiaxial stress measure ($P_{11}+P_{22}$) obtained using ML predicted parameters and $\mathbf{P}=J\,{\varvec{\sigma }}\,\mathbf{F}^{-T}$ formula, was in excellent agreement with the corresponding stress measure from equibiaxial experiments (Fig. 11), indicating that ML parameters predicated from an organ-level measurement (EDPVR) can well describe tissue-level passive stress-stretch relation of myocardium.

### ML prediction for human example

**Figure 12:**
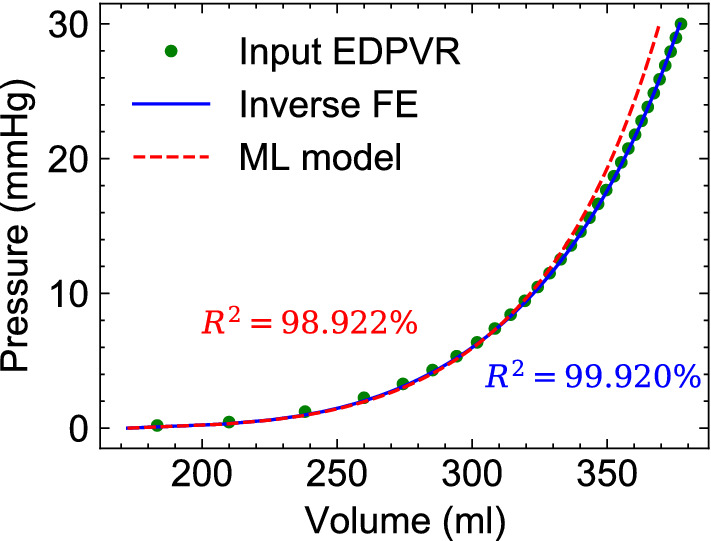
Comparisons between ML model and inverse FE model fits to the target EDPVR. The EDPVR was generated using Klotz’s approach and patient-specific imaging data for a mitral-valve prolapse (MVP) patient. A population average end-diastolic pressure for MVP patients was used to generate the target EDPVR.

The predicted values using ML were in good agreement with those from the inverse FE modeling (Table 5). The EDPVR generated via forward FE simulation using ML-predicted properties fit the input human EDPVR sufficiently well, although poorer than the fit by the inverse FE prediction (Fig. 12). The slight disagreement at larger pressures in the EDPVR is hypothesized to be due to using a (human) patient-specific heart geometry while all the training geometries were from rodents. This discrepancy can be certainly addressed by enriching our training database with anatomical data from large animal and humans.

**Table 5:** ML model prediction of myocardial properties for the CMR-reconstructed human heart compared to the inverse FE model estimations.

Human case study	$a_f\;(kPa)$	$b_f$
Inverse-FE	34.261	11.275
ML model	33.492	12.501

### Feature importance

**Figure 13:**
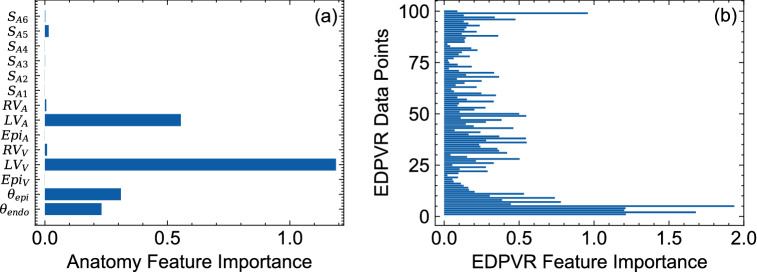
Permutation feature importance results for (**a**) geometric and fiber orientation features and (**b**) EDPVR features. EDPVR features consisted of 100 LV volume data points corresponding to the LV pressure values ranging from 0 to 30 mmHg with a fixed increment.

The results of our permutation feature importance studies indicated that the volume of the LV had the highest importance score among all the geometric features followed by the endocardial area of the LV (Fig. 13a). Other geometric features, including surface areas of short-axis slices, exhibited minimal importance in the predictive power of the ML model (Fig. 13a). The fiber orientation features were found to be important as well, although expectedly with lower scores compared to those of LV volume and area (Fig. 13a). However, EDPVR features, particularly EDPVR points within the bottom and top 10% of the pressure range, had the largest importance score, even up to more than two times larger than LV volume. The relatively higher scores for the low and high pressure regimes in the EDPVR expectedly implies that these portions of the curve are critical to reliably estimate the properties $a_f$ and $b_f$, representing the overall stiffness and the curvature of the EDPVR.

To further investigate the implications of the feature importance study for the relative importance of geometric features, we removed all the geometric features from the 2,500 example dataset except the LV volume and area. A final MFNN network structure with the same hyper-parameters was trained using the updated dataset. Removing the non-contributing or redundant geometric features (i.e., those with low importance score) and reducing them to LV volume and area significantly improved the predictive capability for $b_f$ and slightly reduced the errors for $a_f$ as well (Tables 6 versus 1).

**Figure 14:**
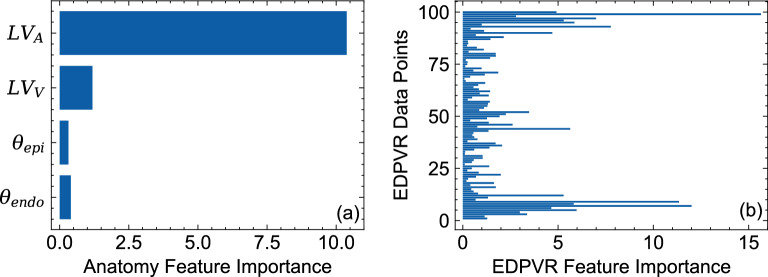
Permutation feature importance results, after removing geometric features with low “importance” score, for (**a**) remaining geometric and fiber orientation features and (**b**) EDPVR features. EDPVR features consisted of 100 LV volume data points corresponding to the LV pressure values ranging from 0 to 30 mmHg with a fixed increment.

We performed a permutation feature importance study for the updated ML model as well (Fig. 14). Contrary to the results of the feature importance study for the previous ML model (Fig. 13) (that included all the geometric features), LV area found to have the highest important score for the updated model followed by LV volume and fiber orientation features, respectively. This is an important observation implying that the LV area is a very critical geometric feature in the absence of short-axis slice features whereas the LV volume was a more important geometric feature once the short-axis slices were present in the training dataset. EDPVR feature importance maintained a similar pattern in the updated model (Fig. 14b) with the highest importance score occurring at low and high pressure ranges. All considered, the LV volume, the LV area, the fiber angles at epi- and endocardial surfaces, and the EDPVR points were found to be sufficient to predict $a_f$ and $b_f$ as myocardial properties.

**Table 6:** Improvement of ML prediction errors after removing geometric features with low “importance” score and using the LV volume and endocardial surface area as the only geometric features.

	MAE	NMAE, $\%$	$R^2$, $\%$
$a_f$	1.241 (kPa)	1.258	99.679
$b_f$	0.711	3.598	96.240

## Discussion

In this paper, we developed a ML model for patient-specific in-vivo estimation of myocardial stiffness from EDPVR and demonstrated its high accuracy. To the best of our knowledge, this is the first study in which a ML model was used as a surrogate for the entire inverse FE process to identify myocardium material properties. The proposed trained ML model was evaluated on a testing dataset not used for training/validation. Minor discrepancies (with NMAE about 1% to 3.5%) were achieved between the actual and ML-predicted myocardial properties. It was further verified that even for the worst prediction cases, the fiber stress obtained with ML-predicted parameters can fit the actual stresses with high accuracy ($R^2>99.9\%$). Furthermore, in addition to the feasibility of the ML model for clinical application, the model exhibited a strong predictive power in our evaluation against two subject-specific measurements not included in training/testing data. The myocardial properties predicted by the model for rat-specific data were in excellent agreement with those estimated from ex-vivo equibiaxial testing of the rat-specific LVFW specimen. The ML model trained using rat heart geometries was still able to predict the myocardial properties for a human heart reasonably close FE inverse mode estimation.

The identification of cardiac tissue-level properties from organ-level measurement are commonly addressed using inverse FE methods^5,8,9,11^. However, the time-consuming procedures involved in these methods, ranging from geometry reconstruction and meshing to iterative FE simulations needed for optimization, significantly limits their efficiency and hampers their use in time-sensitive clinical applications. On the contrary, the proposed ML model, trained on the FE simulation, offers the estimation of myocardial properties directly from geometric, architectural, and EDPVR features that could be feasibly calculated from CMR or CT acquisitions and hemodynamic assessment without the need to advanced computational infrastructure such as FE solvers. It is worth noting that forward FE simulations used to generate training/validation and testing datasets took several days using Abaqus solver on high-performance computing clusters^48,49^ whereas the trained ML model makes predictions within one second.

The geometric features were designed to be compatible with standard cardiac imaging protocols acquiring heart geometry on short-axis planes that are perpendicular to the LV long axis. The features can be exactly calculated from reconstructed geometry, or approximated directly from CMR DICOM images using existing automatic segmentation tools. Some of the features, including LV volume, can be alternatively obtained using echocardiography-based or catheter-based hemodynamic assessment. The EDPVR can be estimated via the Klotz approach by a single-point measurement (typically, the LV pressure and volume at ED). These features can then be used as an array of inputs to the trained and ready-to-use ML model to predict the two output myocardial properties.

Once the ML model is trained, it can be used to make predictions instantaneously and repeatedly. This enables the in-vivo and real-time estimation of patient-specific myocardial properties and offers the inclusion of these properties as tissue-level biomarkers. Such biomarkers provide important information incremental to organ-level measurements (including routine CMR and hemodynamic assessments) and ultimately enhancing the prognosis in heart diseases involving myocardial remodeling. While EDPVR is a measurement of “chamber” stiffness that contains information about tissue-level behavior, it is confounded by anatomy and loading. Our ML inverse model is able to deconvolute the effects of anatomy and loading and estimates intrinsic myocardial properties.

One of the important aspects of our work was to account for the effect of anatomy on the myocardial stiffness prediction and include a wide variety of anatomical characteristic features in our datasets encapsulating both morphology and size of the heart chambers. Our feature importance analysis, indeed, confirmed the importance of geometric features, particularly, LV volume and LV endocardial area, in estimating myocardial properties. Moreover, a broad range of possibilities in transmural fiber orientation variation were included in the training. Again, permutation feature importance technique underscored the knowledge of fiber distribution for predicting myocardial properties although the fiber data was found to be less significant compared to geometric features. Overall, our procedure identified the geometric and architectural information that are sufficient to estimate subject-specific myocardial properties from chamber-level pressure-volume measurements.

Our ML model is not tied to a particular form of the constitutive model for myocardium. The ML learning can be trained on the full H-O model including additional terms characterizing the behavior of the tissue in sheet directions and the coupling with the fiber direction. However, this will lead to an over-parameterized inverse model problem if EDPVR is the only given information on the organ-level loading response. Indeed, EDPVR reflects the chamber behavior under monotonic pressurization (which is only a specific loading condition in the entire loading space), and therefore, the number of myocardial properties that could be estimated from EDPVR remain limited. This constituted our rationale to reduce the parameters to $\{a_f,\ b_f\}$ to minimize potential parameter identifiability issues stemming from different sets of parameters producing similar EDPVR curves. The identification of additional parameters will be possible by including myocardial response under other loading conditions. Potential in-vivo data includes myocardial strain measurements using cine CMR or cardiac CT together with catheter-based intra-ventricular pressure measurements, and examples of ex-vivo inputs includes myocardial stress-strain data under various multi-axial loading conditions. However, given the goal of estimating myocardial properties with minimal in-vivo clinical data (e.g., $P_{ED}$ and $V_{ED}$), $a_f$ and $b_f$ represent appropriate and sufficient parameters characterizing the myocardial in the fiber direction.

The feature importance results consistently indicated that the fiber orientation is an influential input in myocardial properties estimation. This observation is consistent with previous parametric studies investigating the effects of fiber orientation on the EDPVR behavior^50^. Also, this underscores the importance of current research efforts in the development of cardiac imaging protocols to feasibly estimate myofiber orientation, including diffusion tensor imaging and optical coherence tomography. In this regard, our fiber features are only fiber angles at endo- and epicardial surfaces, designed for convenience in measurement as diffusion tensor imaging sequences are currently able to estimate these features in the left ventricle.

Limitations of this study included the use of a relatively simple material model with two unknowns ($a_f$ and $b_f$) that were assumed to be the same everywhere in the biventricular heart. Our objective was to use ML to estimate the *effective* passive stiffness of the myocardium with EDPVR being the only kinematic input. Additional regional kinematic inputs, such as cardiac strains^51,52^, can be used to estimate higher-fidelity properties^53,54^ and consider estimating heterogeneous myocardial properties prevalent in myocardial infraction. The synthetically-generated part of our biventricular geometry dataset preserve the topology of the original 25 image-based hearts and proportionally scale the thickness in the right and left ventricle independently. Additional image-based geometries can improve the heterogeneity of our anatomical dataset and increase the predictive capability of the ML model. Despite significant potential of properly-trained ML models for estimating myocardial properties, fully patient-specific approaches still remain valuable especially when accounting for all the individual anatomical, architectural, and loading data is essential to estimate the properties of interest.

In conclusion, this study has demonstrated that a ML model based on multilayer feed forward neural network can accurately predict myocardial properties using routinely available cardiac imaging and hemodynamic measures and replace computationally-expensive, clinically-unfeasible FE inverse problems. The geometric features (including LV endocardial area) can be approximated from segmented cardiac images without the need for full anatomical reconstruction. EDPVR and architectural features can be also directly measured from hemodynamic assessment and diffusion tenor imaging, respectively. Overall, this study has facilitated the clinical workflow to include the mechanical properties of the myocardium as an additional biomarker to traditional organ-level indices for improved diagnosis, prognosis, and therapeutics of cardiac disease involving myocardial remodeling.

## Supplementary Information

Supplementary Information.

## Data Availability

The data that support the findings of this study are available from the corresponding author, R.A., upon request.
