# Supplementary material for: A machine learning model to estimate myocardial stiffness from EDPVR
Source: Sci Rep. 2022 Mar 31;12:5433. doi: 10.1038/s41598-022-09128-6 (PMC8971532; doi:10.1038/s41598-022-09128-6)
Supplement: Supplementary file 1 — Supplementary Information. [file 41598_2022_9128_MOESM1_ESM.pdf]

Supplementary Information

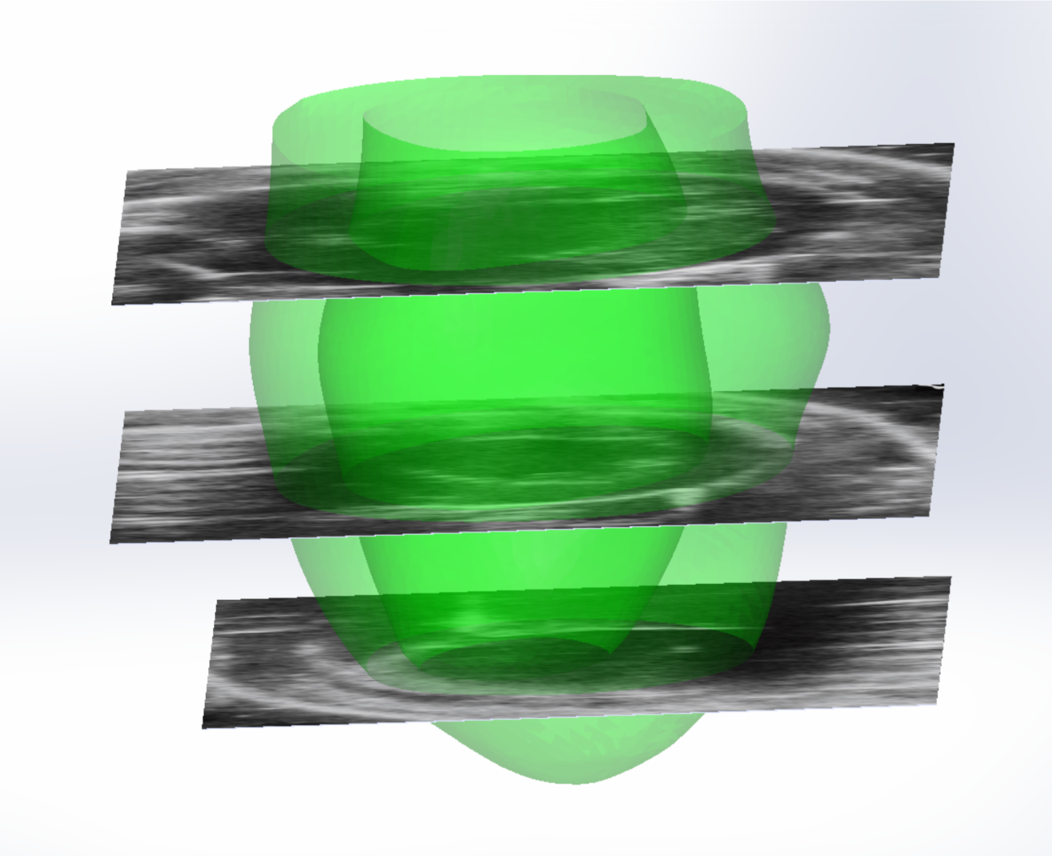

Supplementary Figure 1. Reconstructed LV geometry from short-axis echocardiography slices to estimate geometric features.

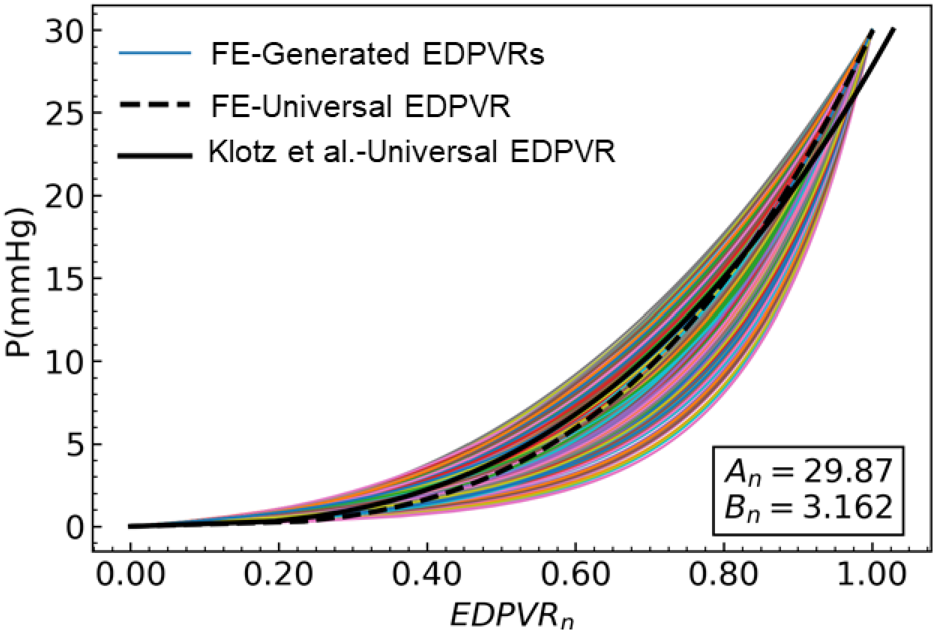

Supplementary Figure 2. Universal EDPVR calculated by normalizing 2,500 individual EDPVRs (Fig. 7) following the method described in Klotz et al.[1].

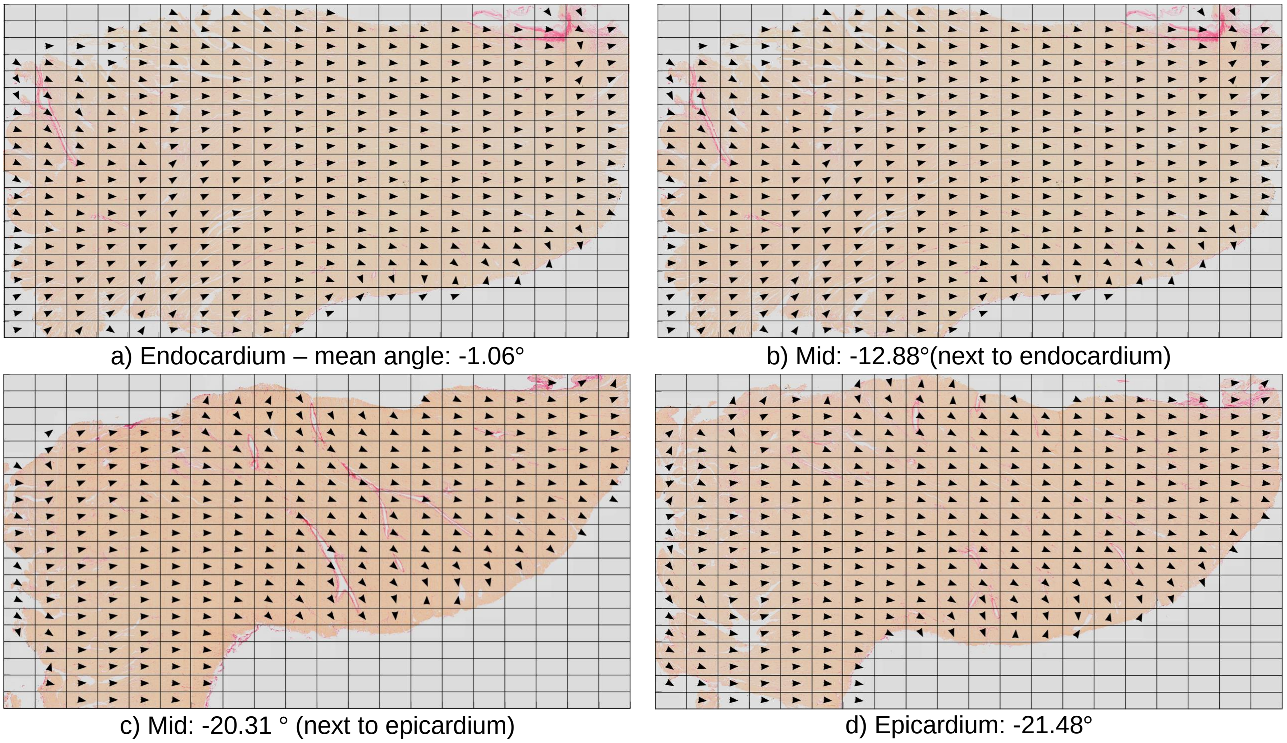

Supplementary Figure 3. Estimation of mean myofiber orientation across the LVFW at four select sections stained by picrosirius red. Black arrows indicate local fiber orientation.

References

1. Klotz, S. *et al.* Single-beat estimation of end-diastolic pressure-volume relationship: a novel method with potential for noninvasive application. *Am. J. Physiol. Circ. Physiol.* **291**, H403–H412 (2006).
